# Rapid Microwave‐Annealing Process of Hybrid Perovskites to Eliminate Miscellaneous Phase for High Performance Photovoltaics

**DOI:** 10.1002/advs.202000480

**Published:** 2020-04-30

**Authors:** Qing Chen, Taotao Ma, Fangfang Wang, You Liu, Sizhou Liu, Jungan Wang, Zhengchun Cheng, Qing Chang, Rong Yang, Wenchao Huang, Lin Wang, Tianshi Qin, Wei Huang

**Affiliations:** ^1^ Institute of Advanced Materials (IAM) and Key Laboratory of Flexible Electronics (KLOFE) Nanjing Tech University (NanjingTech) 5 Xinmofan Road Nanjing 210009 China; ^2^ Department of Materials Science and Engineering Monash University Wellington Road Clayton VIC 3800 Australia; ^3^ Shaanxi Institute of Flexible Electronics (SIFE) Northwestern Polytechnical University (NPU) 127 West Youyi Road Xi'an Shaanxi 710072 China

**Keywords:** broad tolerance window, eliminate miscellaneous phase, microwave annealing process, perovskite solar cells, rapid annealing period

## Abstract

Rapid processing technologies of perovskite solar cells (PSCs) offer an exciting approach to raise the rate of production. Herein, a rapid microwave‐annealing process (MAP) is reported to replace the traditional hotplate annealing process (HAP) and the processing period of perovskite is reduced to less than 1 min. Benefiting from the penetrability and simultaneity of microwave irradiation, the MAP method can effectively eliminate miscellaneous phases and thus achieve >1 µm large‐size crystal grains in perovskite films. These MAP treated perovskite films exhibit pure crystalline phase, long charge‐carrier lifetime, and low defect density, which can substantially improve the PSC efficiency without requiring an additional enhancer/passivation layer. The inverted planar PSCs present enhanced power conversion efficiency from 18.33% (HAP) to 21.59% (MAP) and good stability of >1000 h lifetime without encapsulation under ambient conditions. In addition, MAP can be applied to a large‐size (10 cm × 10 cm) perovskite film fabrication as well as a broader tolerance in environmental temperature and precursor concentration, making it a reliable method for repeatably practical fabrication of perovskite photovoltaics.

## Graphical abstract

Microwave irradiation can accelerate the annealing period of perovskite films from the minute to second scale. The microwave annealed perovskite possesses >1 µm crystal grain size, eliminates miscellaneous phases, and facilitates >21% power‐conversion efficiency.

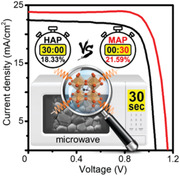

Metal‐halide perovskites have been tremendously developed over the past several years because they can offer the promise of easy fabrication, low‐cost solution‐processability, flexible substrate compatibility, broad bandgap tunability, and integration possibility of tandem multijunction architecture.^[^ ^1^, ^2^ ^]^ Furthermore, perovskite solar cells (PSCs) have already achieved very impressive power conversion efficiencies (PCEs) exceeding 25%.^[^ ^3^ ^]^ This high‐speed enhancement can be attributed to the excellent intrinsic properties of perovskite materials, such as extremely high absorption coefficient and ultralong charge carrier diffusion distance, given by the unique 3D framework of perovskite polycrystals.^[^ ^4^ ^]^ In order to obtain high efficiency and good stability PSCs, great research efforts have been devoted for optimizing the device architecture, deposition techniques as well as regulating the perovskite composition.^[^ ^5^ ^]^ The essential strategy is fabricating high‐quality perovskite film with desirable properties, such as high crystallinity, uniformity, low defect density, and good coverage.^[^ ^6^ ^]^ Therefore, different methods on preparing perovskite films have been proposed, such as one or two sequential spin‐coating deposition,^[^ ^7^ ^]^ vacuum flash‐assisted solution process,^[^ ^8^ ^]^ chemical vapor‐assisted solution process,^[^ ^9^ ^]^ antisolvent assisted crystallization,^[^ ^10^ ^]^ solution‐processed secondary growth,^[^ ^11^ ^]^ self‐seeding growth,^[^ ^12^ ^]^ and cryo‐controlled nucleation technique.^[^ ^13^ ^]^ Among these methods, most of film formation techniques require post‐treatment by thermal annealing to promote further growth and crystallization of perovskite crystals. It is well known that the annealing mechanism of the traditional hotplate‐annealing process (HAP) (**Figure** **1**a) is heat conduction, which transfers heat flow from the bottom hotplate into perovskite film. It thus leads to a nonuniform temperature distribution from bottom to top and inhomogeneous nucleation of perovskite polycrystals. In addition, the processing period by using traditional HAP usually requires dozens of minutes, during this slow annealing process, methylammonium halides in mixed‐ion perovskites are thermodynamically degradable to methylamine, which is a highly volatile component with very low boiling point at −6 °C.^[^ ^14^ ^]^ The degradation and evaporation of methylammonium halides lead to the formation of miscellaneous phase of perovskites^[^ ^15^ ^]^ and thus reduce their PSC performances.^[^ ^2^ ^]^ Therefore, developing a rapid annealing method to eliminate miscellaneous phase in mixed‐ion perovskites is indispensable to the realization of highly efficient PSCs for practical deployment.

**Figure 1:**
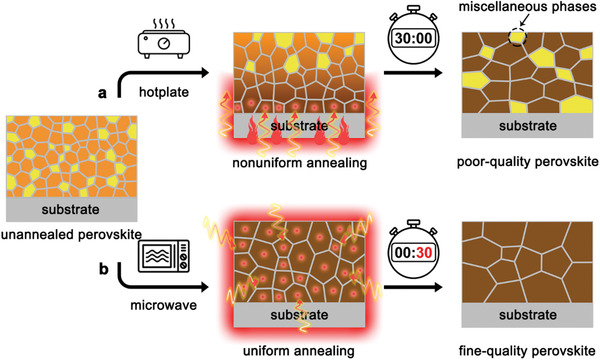
Illustrations of possible phase transition processes in hybrid perovskite films via different annealing methods. a) The hotplate‐annealing process leads to a nonuniform temperature distribution from bottom to top, which makes the inhomogeneous nucleation of perovskite polycrystals, in meanwhile, long annealing period induces the formation of miscellaneous phases. b) The microwave‐annealing process can provide uniform annealing environment all‐around perovskite film, benefitting to accelerate processing duration and achieve fine‐quality perovskite film without miscellaneous phases.

Microwave processing technology has been applied in inorganic and organic synthesis for specific advantages like rapid heating, uniform heating, volumetric heating, power consumption saving, morphology optimizing, and mechanical property improving.^[^ ^16^ ^]^ By using this new generation heating technology, electromagnetic field directly delivers energy to materials though dipolar polarization and ionic conduction, resulting in a significant acceleration on reaction duration: from day‐hour scales to minute‐second scales.^[^ ^17^ ^]^ Therefore, microwave‐assisted synthesis has been commonly used in preparing inorganic metal‐oxide perovskites which were traditionally synthesized in solid‐state at ultrahigh temperature up to >1000 °C for extended periods.^[^ ^18^ ^]^ Recently the microwave synthesis has been extended in preparation of all‐inorganic perovskite nanocrystals for light‐emitting diodes^[^ ^19^ ^]^ and metal‐oxide nanocrystals as electron‐transporting layers for photovoltaic devices.^[^ ^20^ ^]^ However, high performance perovskite solar cell with >20% PCE and >1000 h stability by using microwave irradiation in a facile and reproducible way is quite scarce.

In this work, as shown in Figure 1b, we have developed a rapid and controllable microwave‐annealing process (MAP), which could enormously reduce the duration of treatment to less than 1 min and ensure the formation micrometer‐sized perovskite crystals without any miscellaneous phase. These fine‐quality perovskite thin films are attributed to the advantage of MAP mechanism, which directly converse microwave irradiation energy internally within perovskite in a molecular level and provide a uniform heating diffusion all around perovskite film. Therefore, the MAP method can effectively prevent the decomposition and evaporation of organic components, eliminate miscellaneous phase in hybrid perovskites, and finally promote the formation of high‐quality perovskite films with pure crystalline phase, long charge‐carrier lifetime, and low defect density. Using mixed‐ion perovskite, the PCE of inverted planar p‐i‐n PSCs increases from 18.33% (HAP) to 21.59% (MAP). And the unencapsulated MAP device exhibits only 10% loss of its initial PCE over 1000 h storage under ambient condition.

In order to investigate the different annealing mechanisms of MAP and HAP and thereof effects on the quality of perovskite films, ex situ scanning electron microscopy (SEM) (**Figure** **2**a) and corresponding X‐ray diffraction (XRD) (Figure 2b) measurements were carried out to record the real‐time morphology evolution and phase transformation in MAP treated perovskite films during different annealing periods, using the optimal HAP treated perovskite films as control. Typical Cs_0.05_FA_0.80_MA_0.15_PbI_2.55_Br_0.45_ perovskite films were prepared by one‐step spin‐coating method. The top‐view SEM image of unannealed perovskite films showed mesophase crystal grains containing residual solvents and some miscellaneous precursor phases. After annealing by hotplate at 100 °C for 30 min (the optimal HAP time as control, denoted as HAP‐30m, similarly hereinafter), the crystal grains of HAP‐30m followed a normal grain growth progress where the average grain size increased to about 300–500 nm. However, the grain size of perovskite was not uniform, and many small miscellaneous grains were distributed in the grain boundaries of perovskite polycrystals, due to the disordered growth in perovskite crystallinity and the formation of PbI_2_ after long‐time annealing, which was also confirmed by XRD measurement. The diffraction peak displayed at 12.7° was corresponding to the (001) lattice of a photoinactive miscellaneous PbI_2._ Furthermore, perovskite films treated by MAP at 500 W during different annealing periods (from 10 to 60 s, denoted as MAP‐10s to MAP‐60s, similarly hereinafter) were studied by time‐dependent ex situ SEM and XRD. During the properly annealing periods (10–30 s), microwaves acted as high frequency electric fields and heated the polar solvent molecules (*N*,*N*‐dimethylformamide (DMF), dimethyl sulfoxide (DMSO), and ethyl acetate (EA)) remaining in the perovskite films. The grain size of the MAP perovskite films rapidly increased from 200–400 nm (MAP‐10s), and 400–700 nm (MAP‐20s) to 700–1200 nm (MAP‐30s), possibly due to the digestive ripening effect of residual DMF/DMSO in film, which promoted homogeneous nucleation and thereby enlarged the crystal grain size.^[^ ^21^ ^]^ In addition, by using MAP method, the average grain size increased uniformly in all directions of the films owing to the penetrability and simultaneity of microwave irradiation. It was worth noting that MAP method yielded homogenous films without any miscellaneous phases during 30 s annealing, which were confirmed by both ex situ SEM and XRD patterns. Nevertheless, when the MAP period was further increasing to 40–60 s, the overannealed perovskite films evidently demonstrated miscellaneous phases in SEM images from large‐sized needle‐shape (MAP‐40s), to middle‐sized sheet‐shape (MAP‐50s), and then to small‐sized flake‐fragments (MAP‐60s). These morphological transformations might be attributed to the progressive evaporation of organic components, resulting in excess of lead halides, which were further reconfirmed by the intensity enhancement of diffraction PbI_2_ peak in their corresponding XRD spectra. Moreover, we monitored the steady‐state photoluminescence (PL) spectra (Figure S1, Supporting Information) and fitted values of time‐resolved photoluminescence (TRPL) (Table S1, Supporting Information) of perovskite films treated by MAP and HAP in different annealing periods. Figure 2c reveals the tendency of normalized PL intensity at 771 nm and calculated average PL decay time (*τ*_avg_). We found that both PL intensities and decay times were obviously increased from MAP‐10s to MAP‐30s, indicating that quality of perovskite polycrystals was continuously improved, thus charge‐carrier recombination in perovskite layer was significantly suppressed owing to their minimized grain boundaries. As expected, from MAP‐40s to MAP‐60s, both PL intensities and decay times began to decrease again, since the decomposition of perovskites increased nonradiative recombination centers, which was in coincidence with XRD and SEM conclusions. Notably, the exciton lifetime of MAP‐30s perovskite films on quartz substrates was obviously extended to 1.4 µs, which was more than four‐times longer than the HAP‐30m counterpart (Figure S2, Supporting Information). By all accounts of SEM, XRD, PL, and TRPL results, we can conclude that MAP‐30s perovskite should possess most optimal film morphology, largest crystal grain size, minimum miscellaneous phases, highest absorbance intensity, maximum wavelength offset, as well as least nonradiative recombination. Therefore, in the next part, we will focus on the comparison between MAP‐30s and controlled HAP‐30m perovskite films, which are denoted as MAP and HAP, similarly hereinafter.

**Figure 2:**
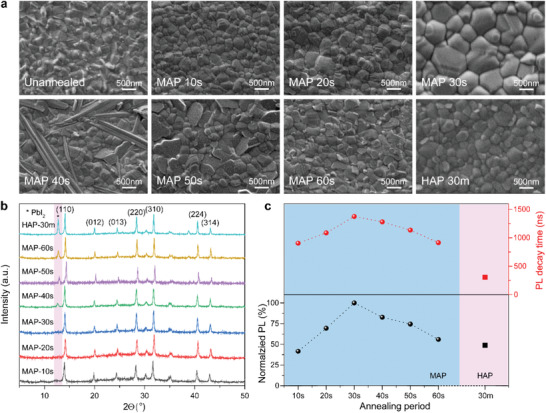
Ex situ characterizations of MAP perovskite films with different annealing periods from 10 to 60 s, using optimal 30 min HAP perovskite as reference. a) Top‐view SEM images, b) XRD spectra, and c) tendency diagram of normalized PL intensities at 771 nm and average PL decay times.

In order to investigate the depth scope of miscellaneous phases in perovskite films, 2D grazing‐incidence wide‐angle X‐ray scattering (2D‐GIWAXS) patterns (**Figure** **3**a) in both bulk‐phase and top‐surface were measured. The controlled HAP perovskite film presented two distinct characteristic peaks located at *q* = 0.9 and 1.0 Å^−1^, which were attributed to the PbI_2_ (001) and perovskite (110) lattices, respectively.^[^ ^22^ ^]^ Noticeably, the MAP showed no signals of PbI_2_ (001) peaks in both bulk‐phase and top‐surface patterns, representing the microwave irradiation acted homogenously and miscellaneous phases controlled effectively in the whole perovskite film. Figure 3b plots photoluminescence quantum efficiencies (PLQEs) of MAP and HAP perovskite films measured in an integrating sphere as a function of excitation power. The data show that the PLQE increases roughly an order of magnitude for MAP film compared to control HAP film across all excitation powers. Importantly, we find at excitation powers which generate carrier densities comparable to 1 sun illumination that the PLQE can be doubly enhanced from ≈3.6% (HAP) to ≈7.3% (MAP), attributed to increasing radiative bimolecular recombination as a result of trap filling.^[^ ^23^ ^]^ It indicated a significant suppression on nonradiative recombination in high‐quality MAP film, that is in corresponding with steady‐state and time‐resolved PL conclusion. The space charge–limited current (SCLC) measurement is utilized to evaluate the dark defect density (*N*_defects_) of MAP and HAP perovskite films and the *N*_defects_ can be obtained from the following equation

(1) $$\begin{array}{c} N_{\mathrm{defects}}=2\frac{V_{\mathrm{TFL}}\varepsilon_{0}\varepsilon_{r}}{ed^{2}} \end{array}$$

Herein, *ε*_0_ is the vacuum permittivity (8.8542 × 10^−14^ F cm^−1^) and *e* is the electric charge (1.602 × 10^−19^ C). *V*_TFL_ is the onset voltage of trap‐filled limit, which can be measured by dark current density–voltage (*J*–*V*) curves using the indium tin oxide (ITO)/perovskite/Au structure. *d* is the thickness of perovskite layers, which is measured by cross‐sectional SEM (Figure S3, Supporting Information). *ε*_r_ is the dielectric constant, which can be used as 28.8 for mixed‐ion perovskite.^[^ ^24^ ^]^ As shown in Figure 3c, the *V*_TFL_ values of mixed‐halide perovskite treated by HAP and MAP as 0.56 and 0.28 V, respectively. According to the above logarithmic *J*–*V* analysis, the calculated *N*_defects_ was reduced by half from 4.72 × 10^15^ cm^−3^ (HAP) to 2.35 × 10^15^ cm^−3^ (MAP). The lower defect density of MAP treated perovskite films indicated in which fewer nonradiative recombination centers were existed due to less miscellaneous phases. The surface energy levels of MAP and HAP perovskite films were estimated by ionized photoelectron spectroscopy (IPS). As shown in Figure 3d, the measured ionization potentials origin from the valence band of MAP and HAP were −5.86 and −5.67 eV, respectively. The energy level change might be due to different ordering of mixed cations and/or halides or dissimilar proportions of miscellaneous phases in perovskite film, that can affect the work function and the interfacial band alignment, which also observed in other report.^[^ ^25^ ^]^ All above measurements indicated that MAP method could achieve a more homogeneous annealing effects to transform precursors into pure perovskite phase without any miscellaneous phase. In meanwhile, the high‐quality MAP perovskite film exhibited pure perovskite phase, long charge‐carrier lifetime, and low defect density.

**Figure 3:**
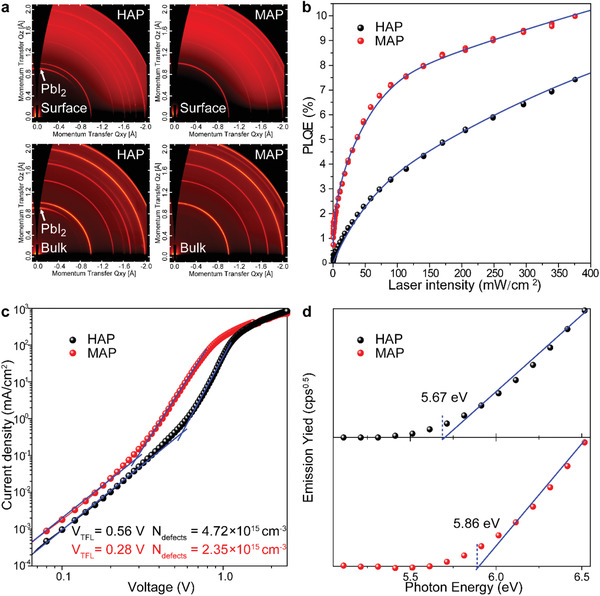
Contrast of structural anisotropy and electrical properties between optimized MAP‐30s and HAP‐30m perovskite films. a) 2D‐GIWAXS patterns of bulk‐phase and top‐surface, b) PLQE spectra, c) dark *J*−*V* curves, and d) IPS spectra.

We evaluated the photovoltaic performances of the MAP and HAP perovskites by fabricating inverted planar heterojunction PSCs with device architecture (**Figure** **4**a): ITO/poly[bis(4‐phenyl)(2,4,6‐trimethylphenyl) amine] (PTAA)/mixed‐ion perovskite (MAP or HAP)/[6,6]‐phenyl‐C61‐butyric acid methyl ester (PC_61_BM)/buckminsterfullerene (C_60_)/bathocuproine (BCP)/silver (Ag). The *J*–*V* curves were measured under simulated AM (air mass) 1.5G (global) illumination at 100 mW cm^−2^. We systematically optimized the processing parameters for the MAP technique (Table S2, Supporting Information). The *J*–*V* curves of MAP‐PSCs exhibited a champion PCE of 21.59% by reverse scan and 21.19% by forward scan with negligible hysteresis (Figure 4b). In contrast to HAP‐PSCs, the MAP‐PSCs exhibited enhancements on both open‐circuit voltage (*V*_oc_) of >0.1 V and short‐circuit current density (*J*_sc_) of >1 mA cm^−2^, which were attributed to lower charge recombination and higher light absorbance. The distribution curves of *V*_oc_, *J*_sc_, fill factor, and PCEs of 20 independent MAP and HAP devices were listed in Figure S4 in the Supporting Information. The monochromatic incident photon‐to‐electron conversion efficiency (IPCE) spectra demonstrated matchable integrated *J*_sc_ values (<4% deviation) to *J*–*V* scan data (Figure 4c). Moreover, the *J*_sc_ value of MAP‐PSC presented an excellent stability by verifying with 600 s evolution of the maximum power point (Figure 4d), whereas the controlled HAP device exhibited 10% PCE loss within initial 180 s. Electrochemical impedance spectroscopy (EIS) measurements were conducted to provide further insight into the charge transport dynamics of devices. Figure 4e shows the dark Nyquist plots with a 1.0 V bias voltage and the corresponding equivalent circuits. Different electronic parameters in Table S3 in the Supporting Information regarded to five components in the equivalent circuit,^[^ ^26^ ^]^ including i) *R*_s_, the series resistance related to electrode connection, ii) *R*_c_, contact the resistance at the interface between perovskite and electron‐ and hole‐transporting layers (ETL and HTL), iii) *R*_rec_, the carrier recombination resistance, iv) *C*_c_, the chemical capacitance, and v) *C*_μ_, the chemical/bulk capacitance of full devices, wherein *C*_c_ and *C*_μ_ were included for more precise fit to equivalent circuits. Compared to the HAP counterpart, MAP device presented not only a lower *R*_c_, arising from more efficient change transport at the ETL/perovskite or perovskite/HTL interface, but also a significantly higher *R*_rec_, clarifying a lower interfacial carrier recombination in MAP device due to its well‐controlled on miscellaneous phases. Considering the same configuration of the device, a lower *R*_c_ and higher *R*_rec_ for MAP‐PSCs were predominately ascribed to the better quality of the perovskite film, resulting in a relative higher *V*_oc._ The fewer defects recombination centers in perovskite films and lower accumulation of charges at the interface between perovskite and charge transporting layers were the dominant reason for the negligible hysteresis of the MAP‐PSCs. The long‐term stability tests of MAP and HAP devices (Figure 4f) were performed in air atmosphere under dark at 25–30 °C and 30–50% relatively humidity (RH) without encapsulation. After >1000 h the MAP device still maintained >92% of the initial PCE, whereas the normalized PCE of control HAP device reduced to 60%.

**Figure 4:**
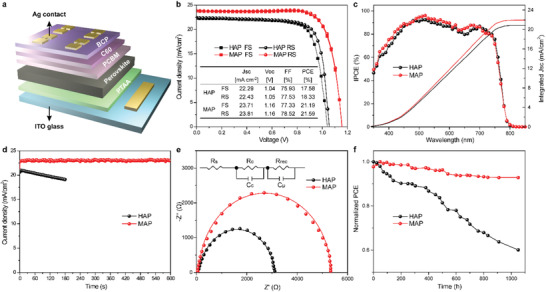
Device architecture and solar cell performance of MAP and HAP devices. a) Device configuration of the planar p‐i‐n perovskite solar cell. b) *J*–*V* characteristic curves of champion devices with reverse and forward scans at rate of 10 mV s^−1^, measured under the solar simulator of AM 1.5G, photovoltaic parameters listed in insert table. c) IPCE spectra and their integrated current densities. d) Steady‐state current density. e) Nyquist plots at a potential bias of 1.0 V, and frequency range from 10 to 10^5^ Hz, in the dark. The inset is the equivalent circuit model. f) Long‐term stability tests in air atmosphere under dark at 25–30 °C and 30–50% RH without encapsulation.

To demonstrate the scalability of our MAP method, one large‐size (10 cm × 10 cm) perovskite film was prepared by MAP with annealing time for 30 s. The photographs were shown in **Figure** **5**a and Figure S5 in the Supporting Information. We measured morphologies and PL intensities at 12 different positions distributed from the center to sides and corners of this large‐sized perovskite film. As atomic force microscopy (AFM) images shown in Figure 5b, the surface of the perovskite films is consecutive and uniform with the average roughness of 23.36 ± 1.86 nm. PL spectra measured from twelve different spots of the large‐sized perovskite films exhibited similar PL intensity as shown in Figure S6 in the Supporting Information. Both AFM and PL measurement strongly indicated a uniform film quality of the large‐sized perovskite film by MAP method. Furthermore, PSCs with active areas of 1.2 cm × 1.2 cm were fabricated by using HAP and MAP. MAP treated PSC still exhibited a high PCE of 18.94% in Figure 5c with an aperture mask size of 1.0 cm × 1.0 cm under AM 1.5G illumination. In contrast, HAP treated PSC with the same size showed only 15.11% due to its poor film‐quality. In addition, the power consumption for MAP can be much less than that of HAP owning to the annealing time is greatly shortened. Therefore, the cost advantage and the potential to expand the device scale of MAP method making it to be a promising technique for the large‐scale fabrication of high‐efficiency PSCs. It is worth noting that in order to prevent uneven internal heating due to the generation of standing waves, the perovskite film was heated in the microwave oven (DAEWOO, KOR‐4A6BR) using a rotating tray, and thus we could obtain high‐efficiency devices repeatably. This problem is also particularly important in the industrial scale‐up.

**Figure 5:**
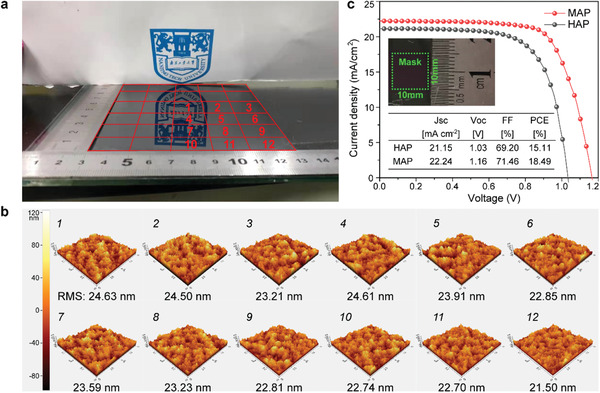
Large‐scale fabrication of perovskite films and PSCs. a) Photograph of 10 cm × 10 cm perovskite film treated by MAP. Twelve different positions located from the center to the corner of this large‐size perovskite film were selected to be further tested. b) AFM topography images and root mean square (RMS) surface roughness from 12 different spots. c) Typical *J*–*V* curves and extracted PV parameters for HAP and MAP‐PSCs with the active areas of 1 cm^2^ as shown by the inset picture.

Furthermore, to verify the operating repeatability of the MAP method at different ambient temperatures, we summarized and contrasted the statistical distributions of PCEs based on MAP‐PSCs and HAP‐PSCs under different device fabrication temperatures such as 22, 26, and 30 °C, respectively. The temperature inside glovebox could be controlled by an air conditioner, which located in an enclosed space together with the whole glovebox as shown in Figure S7 in the Supporting Information. The MAP‐PSCs demonstrated stable PCEs of 18–21% for diverse temperatures on account of >30 independent cells (**Figure** **6**a). Whereas the controlled HAP‐PSCs showed a significant deterioration of PCEs from ≈18% for 22 °C and ≈17% for 26 °C, down to ≈15% for 30 °C in average (Figure 6b). This result denoted that MAP‐PSC performance was steady and insensitive to ambient temperatures, and such a larger operating temperature window would facilitate the transition from laboratory to factory fabrication of PSCs. Moreover, the MAP‐PSCs presented stable PCEs of 19–21% based on varied concentrations of mixed‐ion perovskite precursors from 1.1 to 1.5 m (Figure 6c). In contrast, only 1.3 m concentration of precursors prepared HAP‐PSCs showed normal PCEs of 17–19%, while a decreased 1.1 m or an increased 1.5 m concentrations will result in low PCEs (Figure 6d). This phenomenon signified that novel MAP method could provide a broader tolerance in both concentration and stoichiometry of complex mixed‐ion perovskite precursors, which would be beneficial in reproducing high performance PSCs in large‐scale processes.

**Figure 6:**
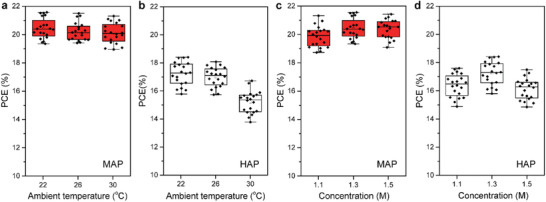
Reproducibilities of MAP and HAP PSCs. a,b) The relation between PCEs and ambient temperatures, 20 devices tested per temperature. c,d) The relation between PCEs and precursor concentrations, 20 devices tested per concentration.

In conclusion, we have developed a rapid, simple, controllable, scalable, energy‐effective, and temperature‐tolerant annealing method—MAP for preparing perovskites, offering a new approach to achieve highly performance planar heterojunction PSCs without requiring an additional enhancer/passivation procedure. MAP method could effectively eliminate the miscellaneous phases and thus achieve fine‐quality mixed‐ion perovskite films which were confirmed by ex situ SEM and XRD analyses. Moreover, both optical measurements including UV–vis, PL, and TRPL spectra and electrical experiments such as SCLC, IPS, and EIS further verified the improvement on film qualities by using novel MAP. By incorporating MAP approach into processing planar p‐i‐n mixed‐ion PSCs, a champion PCE of 21.59% with negligible hysteresis and >1000 h stability without encapsulation were produced. Most importantly, this rapid MAP could significantly accelerate the annealing period of PSCs from minute scale to second scale, in meanwhile, offer a finer uniformity on large‐size (10 cm × 10 cm) film as well as a broader tolerance on environmental temperature and precursor concentration, which are essential to high performance PSCs in repeatable large‐scale fabrications. We thereby consider that our promising MAP method will offer a pathway toward continuous large‐scale fabrication in globally commercializing PSC production in different seasons and regions.

## Experimental Section

##### Materials

Formamidinium iodide (FAI, 99.99%), methylammonium bromine (MABr, 99.99%), lead iodide (PbI_2_, 99.99%), lead bromide (PbBr_2_, 99.99%), BCP (99.99%), and PTAA (*M*_n_ = 3200, *M*_w_ = 4900) were purchased from Xi'an Polymer Light Technology Corp., China. Buckminsterfullerene (C_60_) was purchased from Xiamen Funano New Material Technology Corp., China. [6,6]‐Phenyl‐C61‐butyric acid methyl ester (PC_61_BM, 99.99%) was purchased from Nano‐C Tech., USA. Chlorobenzene (CB), DMF, and DMSO were purchased from Tokyo Chemical Industry. Cesium iodide (CsI) and EA were purchased from Alfa Aesar.

##### Solution Preparation

PTAA solutions were prepared by dissolving 4 mg of polymer into CB (1 mL) and stirring at 25 °C for 2 h. The mixed perovskite precursor Cs_0.05_FA_0.80_MA_0.15_PbI_2.55_Br_0.45_ was prepared by dissolving 1.19 mmol PbI_2_, 0.21 mmol PbBr_2_, 1.12 mmol FAI, and 0.21 mmol MABr in 800 µL cosolvent of DMSO/DMF (1:4, by volume), followed by an addition of 35 µL CsI (2 m in DMSO), to achieve the triple CsFAMA perovskite solution. PC_61_BM solution was prepared by dissolving 10 mg of PC_61_BM into CB (1 mL) and stirring at 25 °C for 12 h.

##### Device Fabrication

ITO glass substrates were cleaned with diluted detergent, deionized water, acetone, and ethanol in sequence in ultrasonic baths for 30 min and then dried using a nitrogen flow. Then the as‐cleaned ITO substrates were treated with UV‐ozone for 30 min. The substrate's size is 1.4 × 2.0 cm^2^. Subsequently, the ITO substrates were transferred to a N_2_‐filled glovebox with H_2_O and O_2_ concentrations of <0.1 ppm. PTAA solutions were spin coated onto the ITO substrates at 6000 rpm (with a ramping rate of 6000 rpm s^−1^) for 30 s and then the samples were heated at 100 °C for 10 min in a N_2_‐filled inert atmosphere. The perovskite layers were deposited by spun a 50 µL mixed perovskite solution at 6000 rpm for 30 s with an accelerated speed of 1000 rpm s^−1^, and 80 µL antisolvents of EA were dropped at the last 5th second. The HAP films were then annealed at 100 °C for 30 min. The MAP films were placed at the center of plate in microwave oven (DAEWOO, KOR‐4A6BR) and processed at maximum output power (500 W) for 10, 20, 30, 40, 50, and 60 s, respectively. After cooling down, 50 µL of PC_61_BM solution was spin coated on the top of perovskite layer at the speed of 1000 rpm (with a ramping rate of 1000 rpm s^−1^) for 45 s. Then, the samples were transferred to a vacuum chamber without being exposed to air. C_60_ (20 nm)/BCP (6 nm) was then thermally evaporated in a vacuum chamber with the base pressure of <5 × 10^−5^ Pa. Finally, a 100 nm silver (Ag) electrode was thermally evaporated through a metal shadow mask with an aperture size of 0.34 cm × 0.34 cm. The 10 cm × 10 cm large‐sized 30s‐MAP perovskite films for AFM and PL measurements were fabricated on PTAA/glass substrates, by spin‐coating 1000 µL mixed perovskite solution at 6000 rpm for 30 s with an accelerated speed of 1000 rpm s^−1^, and 900 µL EA as antisolvent was dropped at the last 5th second. The large‐sized films were then microwave‐annealed at 500 W for 30 s. Before AFM and PL measurements, the 10 cm × 10 cm large‐sized perovskite film was cut into 1.6 cm × 2.0 cm pieces with a glass cutter. PSCs with 1.0 cm^2^ active areas were fabricated by using 30m‐HAP and 30s‐MAP method, respectively, followed by an evaporation of top‐electrodes with mask sizes of 1.2 cm × 1.2 cm. Then *J*–*V* characterizations were measured by using a 1.0 cm × 1.0 cm aperture mask. All the materials, solutions and preparation process for large area perovskite films were the same as small‐size PSCs.

##### Device Characterization

The surface morphologies and microstructures of the perovskite films were investigated using a field‐emission scanning electron microscopy (Zeiss Ultra Plus). The different perovskite films were tested by an X‐ray diffractometer (XRD, Rigaku, SmartLab3KW) using Cu*K*_*α*_ radiation. The optical absorption of the perovskite samples was measured using a UV–vis spectrophotometer (Shimadzu, UV‐1750). The steady‐state PL spectra were obtained using a PL microscopic spectrometer (Hitachi, F‐4600). 2D‐GIWAXS experiments were performed at small/wide‐angle X‐ray scattering (SAXS/WAXS) beamline at the Australian Synchrotron, part of Australian Nuclear Science and Technology Organisation (ANSTO). The TRPL was measured at 780 nm using excitation with a 510 nm light pulse from Delta Flex Fluorescence Lifetime System (Horiba Scientific Com., Japan). The EIS measurements were carried out by an electrochemical‐lab (CH Instruments Ins, CHI760E). The photocurrent density–voltage curves of the perovskite solar cells were measured using a solar simulator (Class AAA, XES‐40S3‐TT, San‐EI Electric, Japan) and a Keithley 2400 source meter. The intensity (100 mW cm^−2^) was calibrated using a standard Si solar cell (Oriel, VLSI standards). All the devices were tested under AM 1.5G sun light (100 mW cm^−2^) with a scan rate of 10 mV s^−1^. PSCs with 1.0 cm^2^ active areas use a shadow mask with an aperture size of 1.0 cm × 1.0 cm. PSCs with 0.1 cm^2^ active areas use a shadow mask with an aperture size of 0.32 cm × 0.32 cm.

## Conflict of Interest

The authors declare no conflict of interest.

## Supporting information

Supporting Information
