# Supplementary material for: Rapid Microwave‐Annealing Process of Hybrid Perovskites to Eliminate Miscellaneous Phase for High Performance Photovoltaics
Source: Adv Sci (Weinh). 2020 Apr 30;7(12):2000480. doi: 10.1002/advs.202000480 (PMC7312440; doi:10.1002/advs.202000480)
Supplement: Supplementary file 1 — Supporting Information [file ADVS-7-2000480-s001.pdf]

Supporting Information

Rapid Microwave-Annealing Process of Hybrid Perovskites to Eliminate Miscellaneous Phase for High Performance Photovoltaics

Qing Chen[1]†, Taotao Ma[1]†, Fangfang Wang[1]†, You Liu[1], Sizhou Liu[1], Jungan Wang[1], Zhengchun Cheng[1], Qing Chang[1], Rong Yang[1], Wenchao Huang[3], Lin Wang[1], Tianshi Qin[1]*, Wei Huang[2]*

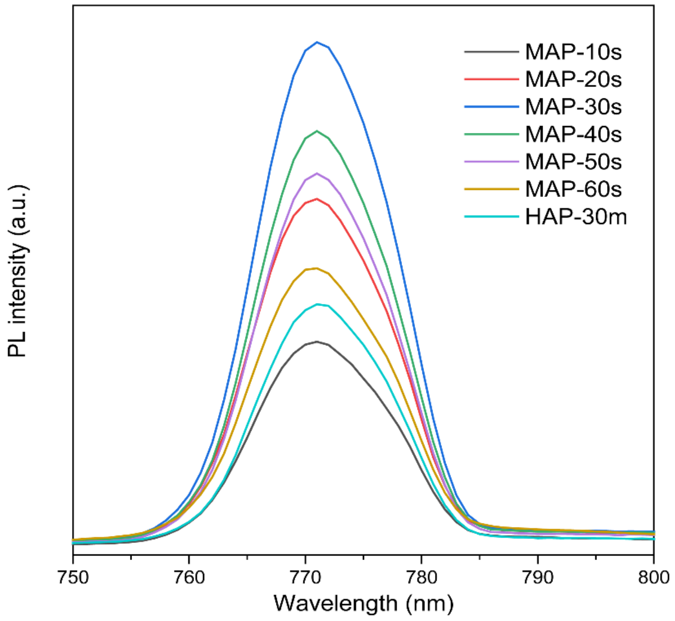

Figure S1 Steady-state PL spectra of MAP and HAP perovskite films with different annealing periods on quartz substrates.

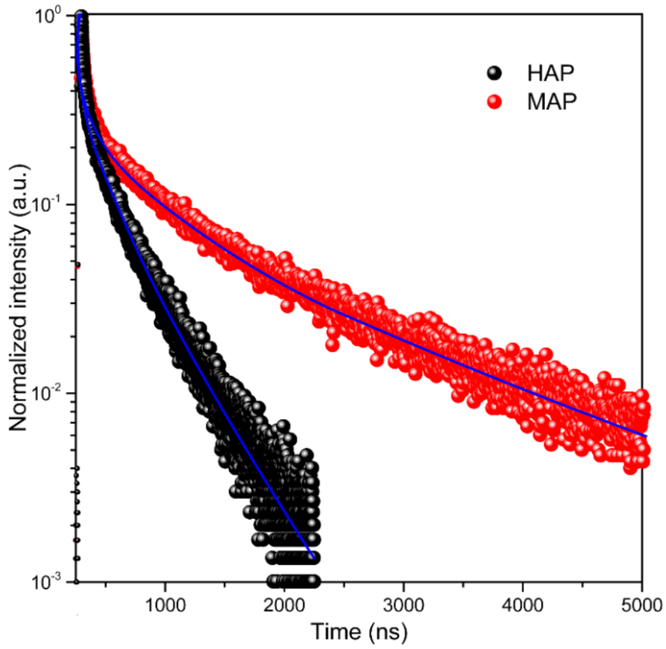

Figure S2 Time-resolved PL spectra of MAP-30s and HAP-30m perovskite films on quartz substrates.

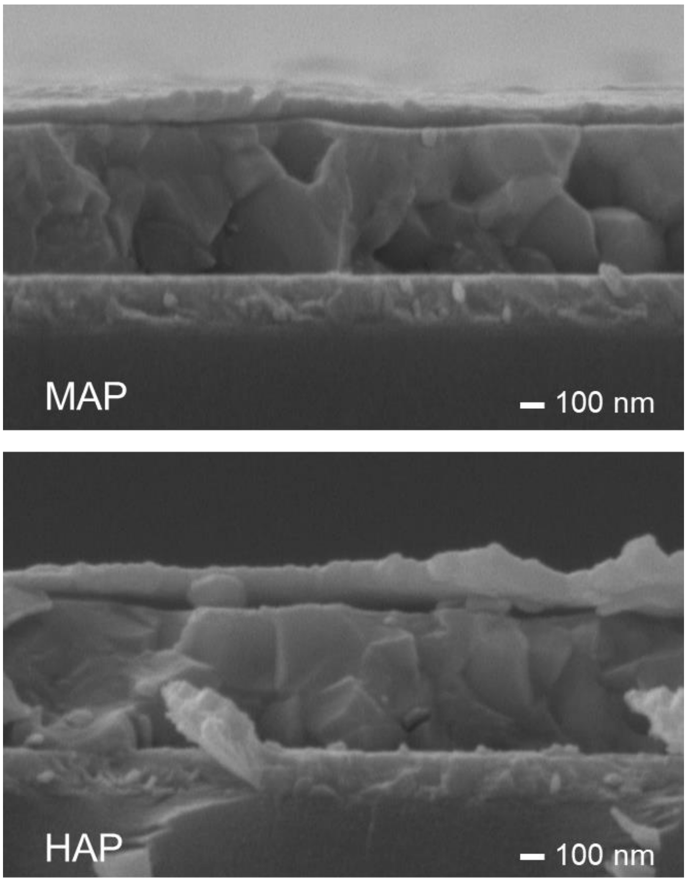

Figure S3 Cross-sectional SEM images of MAP and HAP devices with ITO/perovskite/Au structures for SCLC measurements.

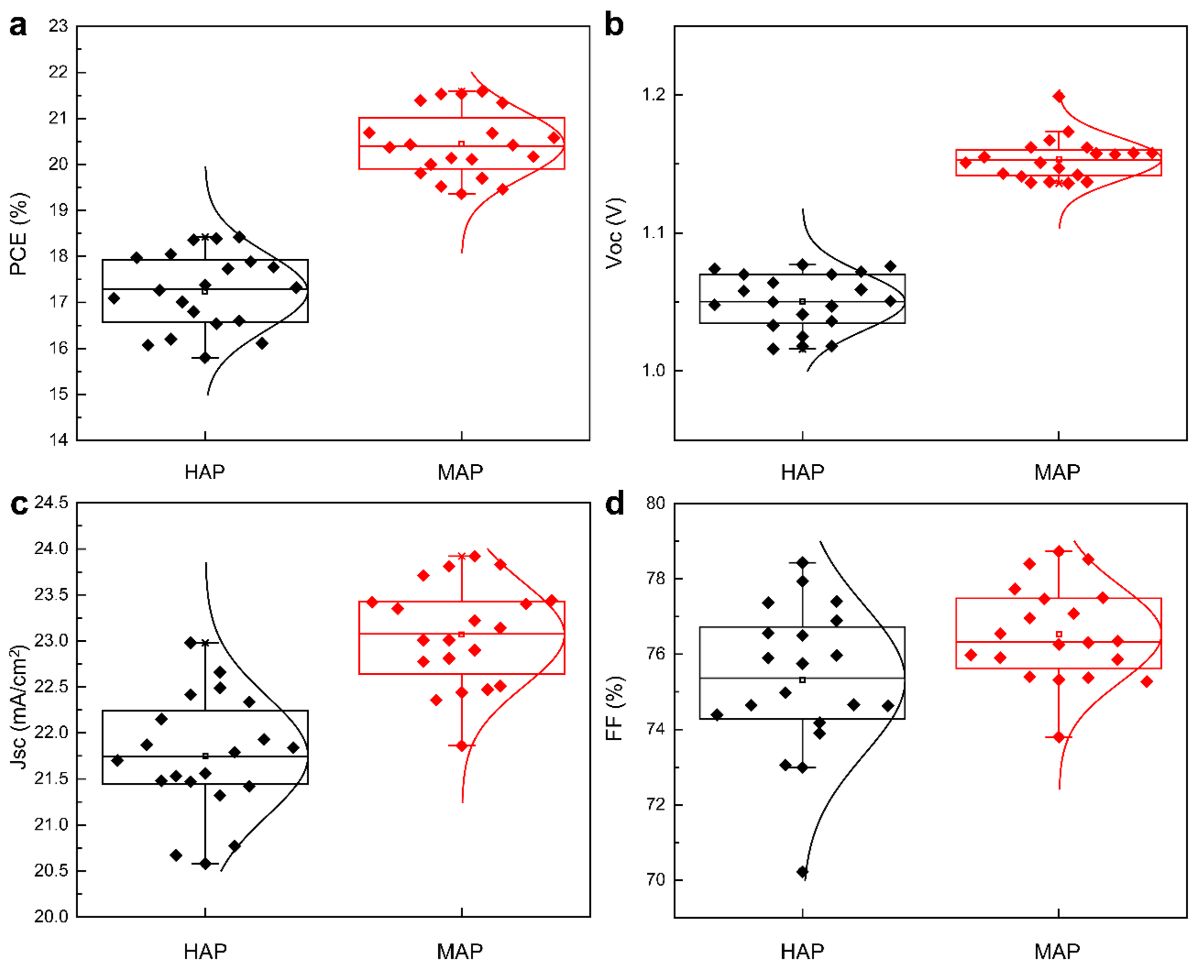

Figure S4 The distribution curves of V_{oc}, J_{sc}, fill factor (*FF*) and PCEs of 20 independent MAP and HAP PSCs.

Table S1 Parameters of the time-resolved photoluminescence (TRPL) spectroscopy of perovskite films on quartz substrates processed by HAP and MAP with different periods, respectively.

	τ_1 [ns]	A_1 [%]	τ_2 [ns]	A_2 [%]	τ_3 [ns]	A_3[%]	τ_{ave}[ns]
HAP-30m	16.98	6.16	127.12	25.91	402.69	67.93	307.53
MAP-10s	29.04	4.90	260.97	23.14	1169.84	71.97	903.74

MAP-20s	100.00	3.30	476.48	39.23	1558.19	57.47	1085.71
MAP-30s	139.62	5.39	667.21	40.84	2034.43	53.78	1374.13
MAP-40s	144.23	4.76	600.91	40.22	1871.77	55.02	1278.39
MAP-50s	85.35	4.84	506.44	38.47	1652.23	56.58	1133.87
MAP-60s	34.20	8.28	367.48	39.01	1455.67	53.71	913.46

The PL decay time and amplitudes are modeled using a biexponential expression:

$$f(t) = \sum_i A_i \, exp\left(-\frac{t}{\tau_i}\right) + K$$

where A_i is the decay amplitude, τ_i is the decay time and K is a constant for the base-line offset. The average PL decay times (τ_{ave}) are further estimated using the τ_i and A_i values from the fitted curve data **Table S1** using:

$$\tau_{ave} = \frac{\sum A_i \tau_i^2}{\sum A_i \tau_i}$$

Table S2 Photovoltaic performance of devices fabricated from different processing periods and different microwave output power efficiencies (microwave oven: DAEWOO, KOR-4A6BR, fixed power source type, output efficiencies set on control panel between 20% and MAX by manufacturer's programing on interval spare times). PV parameters were measured from best performing devices under the standard AM 1.5G illumination. [a)] The output power of MAP is 500 W and temperature of HAP is 100°C. [b)] The MAP period is 30 seconds. [c)] The values in the parentheses are the average PCEs from over 20 devices.

Period [a)]	V_{oc} [V]	J_{sc} [mA/cm^2]	*FF* [%]	PCE [%] [c)]
MAP-10s	1.13 (1.12±0.02)	20.00 (19.59±1.56)	73.38 (70.25±3.95)	16.59 (15.15±1.44)
MAP-20s	1.14 (1.14±0.01)	22.84 (22.06±1.28)	76.23 (73.56±3.57)	19.92 (19.36±0.65)

MAP-30s	1.16 (1.15±0.03)	23.81 (23.06±1.20)	78.52 (76.54±2.74)	21.59 (20.44±1.15)
MAP-40s	1.14 (1.13±0.02)	23.67 (22.65±1.64)	77.83 (75.34±3.18)	20.87 (19.87±1.08)
MAP-50s	1.13 (1.12±0.01)	22.84 (21.86±1.39)	78.48 (75.85±3.26)	20.35 (19.58±0.99)
MAP-60s	1.13 (1.12±0.02)	22.47 (21.52±1.55)	77.96 (74.98±3.52)	19.82 (19.22±0.75)
HAP-30m	1.05 (1.03±0.03)	22.43 (21.23±1.68)	77.53 (74.58±3.19)	18.33 (17.24±1.98)

Output power efficiency [b)]	V_{oc} [V]	J_{sc} [mA/cm2]	*FF* [%]	PCE [%] [c)]
20%	1.15 (1.14±0.02)	18.30 (17.59±1.46)	72.12 (70.13±3.22)	15.17 (14.25±1.13)
40%	1.17 (1.15±0.02)	18.09 (17.66±1.85)	75.26 (73.28±3.29)	15.92 (15.53±0.57)
60%	1.17 (1.15±0.03)	20.55 (20.05±1.87)	75.17 (74.56±3.74)	18.07 (17.69±0.75)
80%	1.17 (1.15±0.02)	22.69 (21.98±1.56)	76.54 (75.66±2.88)	20.31 (19.45±0.98)
MAX	1.16 (1.15±0.03)	23.81 (22.65±1.64)	78.52 (76.54±2.74)	21.59 (20.44±1.15)

Table S3 Fitted values of different electronic parameters from dark Nyquist plots of HAP and MAP devices.

Device	R_S (Ω·cm^2) (Deviations)	R_C (Ω·cm^2) (Deviations)	R_{REC} (Ω·cm^2) (Deviations)	C_C (nF/cm^2) (Deviations)	C_μ (nF/cm^2) (Deviations)
HAP	3.58 (7.67%)	24.32 (5.81%)	291.40 (1.14%)	236.66 (3.63%)	44.00 (1.08%)
MAP	1.83 (9.37%)	15.72 (1.71%)	515.20 (1.93%)	208.48 (3.07%)	40.91 (2.02%)

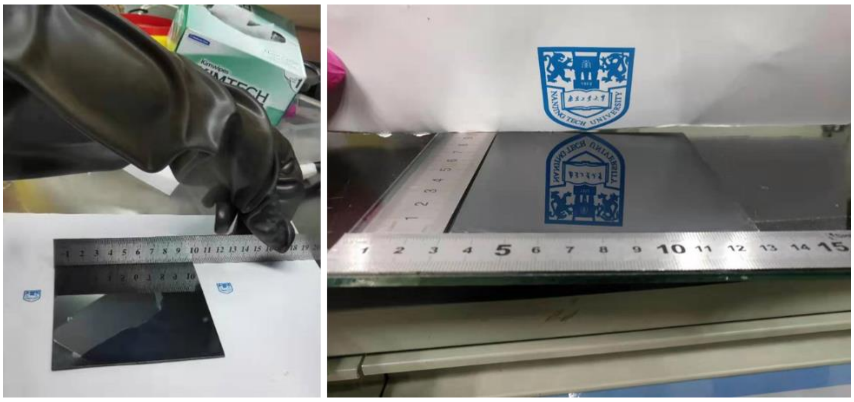

Figure S5 Photograph of the 10mm ×10mm large-scale perovskite films treated by MAP.

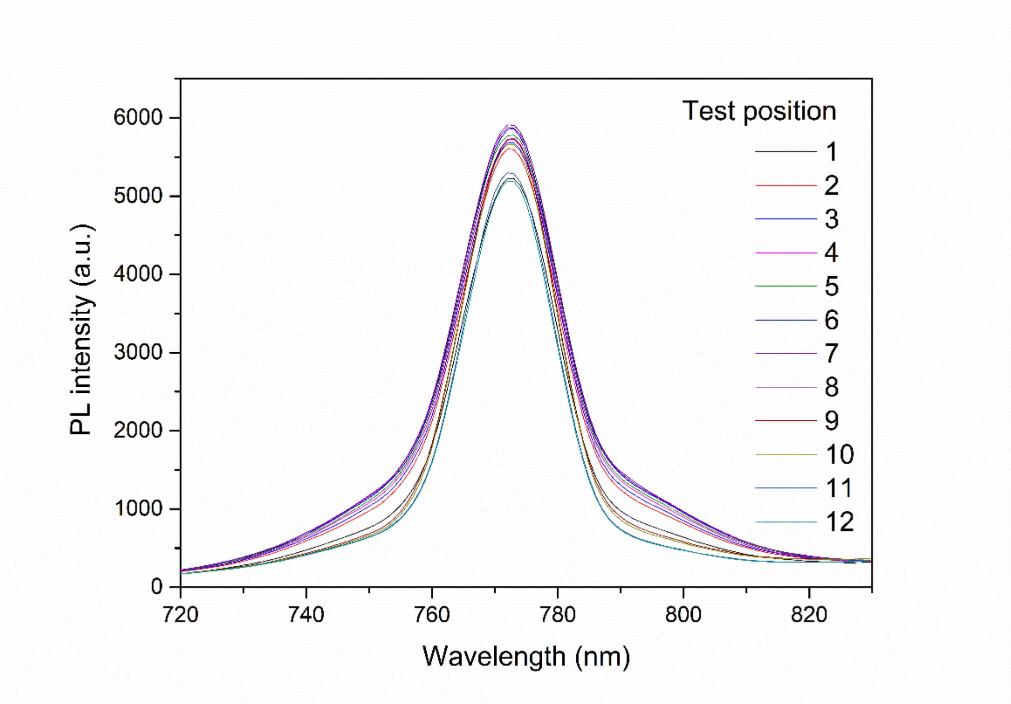

Figure S6 Steady-state PL spectra of MAP perovskite films measured form twelve different spots of the large area perovskite films on glass substrates.

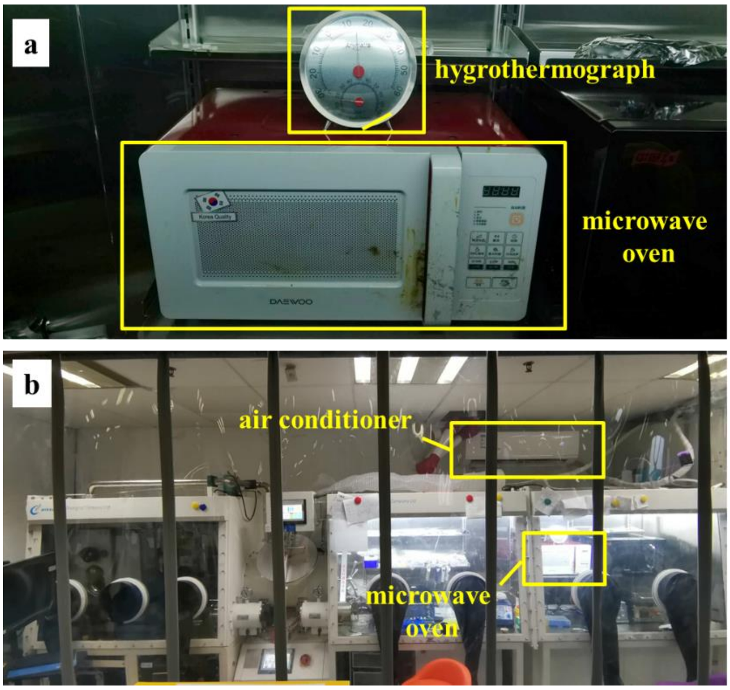

Figure S7. a) The microwave oven in the glovebox; b) The inside temperature of the glovebox controlled by air conditioner in an enclosed space.
